# Personalized Intervention Strategy Based on a Risk Score Generated From Subcutaneous Insertable Cardiac Monitor: Results From Phase 1 of ALLEVIATE‐HF

**DOI:** 10.1161/JAHA.124.035501

**Published:** 2024-10-11

**Authors:** Rami Kahwash, Michael R. Zile, Prasad Chalasani, Barry Bertolet, Laura Gravelin, Muhammad Shahzeb Khan, Jennifer Wehking, Brian Van Dorn, Shantanu Sarkar, Verla Laager, Bart Gerritse, Aimee Laechelt, Javed Butler, the ALLEVIATE‐HF Phase I Investigators

**Affiliations:** ^1^ The Ohio State University Columbus OH USA; ^2^ Medical University of South Carolina Charleston SC USA; ^3^ Ralph H. Johnson Department of Veterans Affairs Health Care System Charleston SC USA; ^4^ Florida Heart Center Fort Pierce FL USA; ^5^ Cardiology Associates Research LLC North Mississippi Medical Center Tupelo MS USA; ^6^ Mount Carmel Health System Columbus OH USA; ^7^ Duke University Medical Center Durham NC USA; ^8^ Medtronic Mounds View MN USA; ^9^ Medtronic Bakken Research Center Maastricht Netherlands; ^10^ Baylor Scott and White Research Institute Dallas TX USA; ^11^ University of Mississippi Medical Center Jackson MS USA

**Keywords:** heart failure, protocolized intervention pathway, remote monitoring, risk metric, Heart Failure

## Abstract

**Background:**

Diagnostic variables from insertable cardiac monitors may be useful in identifying patients at increased risk of heart failure (HF) events. High‐risk alerts must be coupled with interventions to improve outcomes. We aim to assess the safety of a predefined protocolized intervention pathway activated by insertable cardiac monitor high‐risk alerts.

**Methods and Results:**

ALLEVIATE‐HF (Algorithm Using LINQ Sensors for Evaluation and Treatment of Heart Failure) Phase 1 was a randomized interventional study enrolling patients with New York Heart Association class II/III and a recent HF event. A HF risk score based on insertable cardiac monitor diagnostics, including impedance, respiration rate, atrial fibrillation burden, heart rate during atrial fibrillation, heart rate variability, and activity duration, was calculated. A protocolized intervention pathway was activated when high‐risk scores were detected that involved physician‐prescribed nurse‐implemented uptitration of diuretic for 4 days, unless safety rule‐out conditions were met. Interventions could be repeated if high‐risk scores persisted and did not require worsening symptoms. In total, 59 patients were randomized (mean age 68.2±11.8 years; 59.3% male); 67.8% with ejection fraction ≥50%. The mean follow‐up was 11.8±8.1 months. Overall, 146 high‐risk scores were recorded in 33 patients and 118 interventions occurred in 75 (51.4%) high‐risk alerts that did not meet safety rule‐out criteria. There were no serious adverse events and 13 adverse events related to interventions. In patients with symptoms at intervention initiation, symptoms resolved in 37 interventions (80%) and worsened in 8 (17%). In asymptomatic patients, symptoms developed in 3 interventions (7%).

**Conclusions:**

A personalized medication intervention based on insertable cardiac monitor risk score can be safely instituted in patients with HF, irrespective of symptoms.

**Registration:**

URL: https://www.clinicaltrials.gov; Unique Identifier: NCT04452149.

**Nonstandard Abbreviations and Acronyms**
AE: adverse events
ALLEVIATE‐HF: Algorithm Using LINQ Sensors for Evaluation and Treatment of Heart Failure
HFE: heart failure event
ICM: insertable cardiac monitor
MCMS: Medtronic Care Management Services
PRN: *pro re nata* or as needed

**Clinical Perspective**

**What Is New?**

- Subcutaneous insertable cardiac monitors have investigational sensors that measure heart failure (HF) related diagnostics taken by ECG, impedance, and 3‐axis accelerometer and continuously generate a multimeric risk score, with the ability to identify patients at increased risk of developing HF events.
- In Phase 1 of the ALLEVIATE‐HF (Algorithm Using LINQ Sensors for Evaluation and Treatment of Heart Failure) study, we establish the safety of an investigator‐directed, nurse‐executed 4‐day diuretic intensification strategy triggered solely by insertable cardiac monitor high‐risk score in patients at risk of worsening HF, with no serious adverse effects reported after a total of 118 diuretic interventions. Resolution of high‐risk scores was seen in most patients following diuretic interventions, irrespective of the presence or absence of worsening HF symptoms at the time of diuretic intervention initiation.

**What Are the Clinical Implications?**

- Insertable cardiac monitor‐based monitoring strategy provides a minimally invasive remote monitoring alternative for patients with HF who are otherwise not eligible for cardiac implantable electronic devices, and it provides a less invasive approach than direct pulmonary artery hemodynamic sensors, not relying on patients for data transmissibility.
- The article provides a detailed description of a protocolized intervention pathway implemented by centralized HF nursing that bridges the gap between alerts and early interventions and can be safely implanted in response to remote alerts; the ability of insertable cardiac monitors to detect arrhythmias accurately offers several additional advantages in HF management that could potentially improve overall outcomes.

The management of patients with heart failure (HF) in an ambulatory setting is challenging, including identifying patients at high risk of experiencing recurrent HF events. It is also important to intervene early to prevent hospitalization. To address these challenges, subcutaneous insertable cardiac monitors (ICMs) with sensors have been developed.^1^ ICMs measure HF‐related diagnostics through ECG, tissue impedance, and 3‐axis accelerometers in a continuous manner. The ICM implant procedure is minimally invasive and the device can be inserted in office.^2^, ^3^ The ICM remote monitoring capability and the passive nature of data acquisition and transmissibility eliminates dependence on patients to take daily measurements. Also, ICMs provide a comprehensive set of arrhythmia^4^, ^5^, ^6^, ^7^, ^8^ and HF‐related diagnostic parameters that have been validated in predicting worsening HF before symptoms occur.^1^ Although cardiac implantable electronic devices have sensors as well,^9^, ^10^, ^11^, ^12^, ^13^, ^14^, ^15^ their clinical usefulness applies only to patients who meet their implantation criteria, excluding a proportion of patients with HF and reduced ejection fraction, and almost all patients with HF and mildly reduced and preserved ejection fractions.

Previous studies have shown that HF monitoring is effective in improving outcomes when using diagnostics‐guided structured intervention protocols^16^, ^17^, ^18^, ^19^ but not in the absence of an actionable plan.^20^, ^21^, ^22^ This highlights the significance of creating a personalized medication intervention plan that is seamlessly integrated into a standardized workflow and is implemented in response to high‐risk alerts.^23^, ^24^

The purpose of this study was to investigate the safety of a physician‐guided, nurse‐implemented, centralized, and protocolized intervention strategy for HF management using the multiparameter risk score based on diagnostics parameters measured by an ICM, among high‐risk patients with HF enrolled in Phase 1 of the ALLEVIATE‐HF (Algorithm Using LINQ Sensors for Evaluation and Treatment of Heart Failure) study. We also assessed whether the intervention strategy will resolve or prevent the worsening of HF symptoms and will affect the resolution of ICM‐based high HF risk score.

## Methods

### ALLEVIATE‐HF Phase‐1 Study Design

The ALLEVIATE‐HF study (NCT04452149) is a prospective, randomized, blinded multicenter intervention study aimed at assessing the safety and efficacy of protocolized intervention pathways triggered by ICM high‐risk scores in heart failure patients with New York Heart Association class II/III and a recent HF event. The study was conducted in compliance with international ethical and scientific quality standards and the principles of the Declaration of Helsinki. The protocol was approved by all relevant institutional review boards and all patients provided written informed consent before initiation of any study‐specific procedures. The trial had major protocol changes after enrollment started, and it was decided that patients enrolled early would not be included in the main study analyses. This early cohort is considered “Phase 1” and is reported upon here, independently from the main ALLEVIATE‐HF results. The data that support the findings of this study are available from the corresponding author upon reasonable request.

The primary objective of Phase 1 of the study was to characterize the safety of the patient management pathway by assessing the rate of *pro re nata* or as needed (PRN) intervention‐related serious AEs (SAE) and there was no specified safety cutoff. The primary safety objective of Phase 2, defined before the reporting of this Phase 1 data, was also the rate of PRN intervention‐related SAEs with a predefined safety cutoff of 5%. The secondary safety objective for Phase 2 was the rate of Reveal LINQ system‐related or procedure‐related SAEs at 6 months.

The complete inclusion and exclusion criteria for the study are detailed in Table S1. The study was conducted at sites specializing in cardiology. It sought to drive diversity in the steering committee selection and trial participants by selecting sites with access to a diverse population and providing recruitment videos that included ethnic minority patients as well as health care providers. Trial participant demographics were monitored in comparison to the disease mix and investigators were reminded of diversity representation goals at investigator meetings.

Although the primary purpose of Phase 1 was to evaluate the safety and logistical feasibility of the intervention protocol before embarking on the larger Phase 2 randomization study, it was determined additional observation data were needed for algorithm validation.^1^ Therefore, a 2:1 randomization design was chosen to ensure the total observational data collected would be adequate for algorithm validation and to gather blinded device data that could be incorporated into the HF risk score performance.^1^ Patients were randomized to ensure both arms would be comparable but not with the intention to compare the arms. Risk score data obtained from the observation arm during the first 7 months were collected but not used to trigger interventions. Patients were randomized via computer‐generated permutated blocks 2:1 between observation with standard HF management alone and intervention including PRN intervention upon high risk in addition to standard HF management. Site personnel and patients were aware of treatment assignments in phase 1. Those randomized to observation had an observation period that lasted for 7 months postrandomization, after which patients crossed over to the intervention arm per protocol. The sample size of 63 enrolled patients was designed with an assumption that 10% of these patients would eventually withdraw or discontinue study participation.

Data used in the current analysis were collected from (1) patients initially randomized in the intervention arm and (2) patients who were initially randomized to observation and crossed over per protocol to the intervention phase 7 months postrandomization. Hence, the study assessed the safety of interventions in all participants. Risk scores in both arms were collected starting 6 weeks post ICM implant. This was to allow sufficient time for pocket healing and accurate measurement of surrounding tissue bioimpedance.

### Protocolized Intervention Pathway

The ICM used 3 sensors, ECG, accelerometer, and impedance, for multiple measurements that are aggregated into daily measurements of subcutaneous fluid, respiratory rate, atrial fibrillation (AF) burden, ventricular rate during AF, activity duration, nighttime and daytime heart rate, and heart rate variability. Measurements over multiple days were combined into a HF risk score as previously described.^1^ When the risk score crossed a high‐risk threshold, it generated an alert that triggered the protocolized intervention pathway. The alert was received by the team of certified HF nurses at Medtronic Care Management Services (MCMS) who were responsible for implementation of the intervention. The centralized implementation through MCMS ensured that interpretation of diagnostics and interventions were standardized, technical issues with remote transmissions were minimized, and logistical barriers related to medication access were identified and resolved in a timely manner to maximize timely intervention.^21^, ^22^ The roles of MCMS nurses are further described in Data S1.

**Figure 1:**
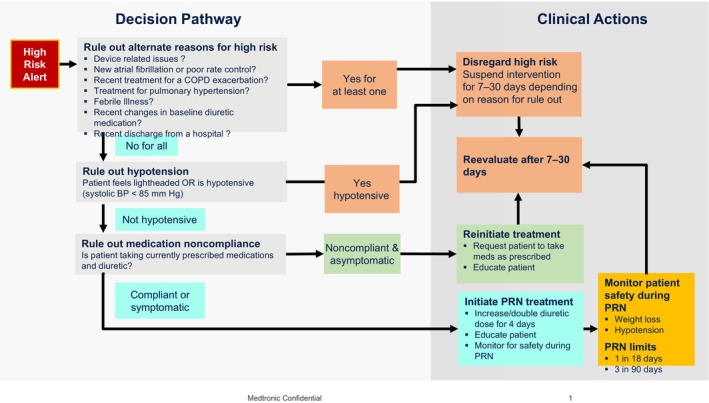
A basic schematic of the HF risk score‐based protocolized intervention pathway. BP indicates blood pressure; COPD, chronic obstructive pulmonarydisease; HF, heart failure; Meds, medications; and PRN, *pro re nata* or as needed.

The schematic of the protocolized intervention pathway is shown in Figure 1. The first step was to evaluate if an intervention should not occur, for example, (1) an alternate medical condition was present that suggested a diagnosis other than HF, (2) recent HF medication changes, (3) logistic barriers to intervention, or (4) other reasons (eg, hospice or rehabilitation care, device‐related problems, protocol mandated intervention suspension, etc.). If a rule‐out condition was met, the intervention pathway was suspended for a specified number of days after which the patient was reevaluated. If an alternate condition suggested that an intervention would be unsafe or otherwise not recommended for PRN interventions (eg, end‐stage kidney disease, continuous inotropic infusions), the case was referred to the steering committee for exclusion from subsequent interventions. If an alternate medical condition was transient (eg, febrile illness, low blood pressure, or recent hospitalization), the intervention pathway was suspended for 7 days. If a recent HF medication change had occurred, the intervention pathway was suspended for 14 days. If the patient was noncompliant with baseline diuretic without worsening symptoms, the patient was encouraged to take the diuretic as prescribed and was reevaluated in 7 days. If the patient was noncompliant and symptomatic, then MCMS nurses encouraged the patient to take their baseline diuretic and initiate the PRN intervention.

Investigators were asked to prespecify safety thresholds for ventricular rate during AF for each patient (eg, >110 beats per minute). When AF contributed to the high‐risk alert and the patient had no history of AF, or when the AF ventricular rate safety threshold was met in a patient with a history of AF, the intervention pathway was suspended until the investigator provided approval to continue the intervention, allowing investigators to initiate AF management per their preferences. If a device issue, for example, device rotation, was identified, the intervention pathway was suspended for 30 days.

If none of the rule‐out criteria were satisfied, MCMS nurses initiated a 4‐day course of PRN medication, which was implemented irrespective of the signs and symptoms of HF. It has been previously reported that diagnostics‐based interventions are also effective in asymptomatic or mildly symptomatic patients.^16^, ^18^ The PRN medication and dosage were decided by investigator discretion at enrollment, were personalized for each patient and could be modified during the study. The protocol was deliberately designed to incorporate a patient‐specific diuretic plan, allowing flexibility in the selection of diuretics such as doubling loop diuretics or adding thiazide diuretics. The decision on which additional PRN diuretics to prescribe and their respective dosages at the time of patient enrollment and modifications throughout the study was entrusted to the health care provider who is most knowledgeable about the patient's previous diuretic needs and response. Specified discontinuation thresholds for a decrease in blood pressure or weight loss during PRN were determined by the investigator to ensure safety. The weight and blood pressure thresholds were monitored remotely and if not available, a symptom questionnaire was obtained. If safety thresholds were met or the patient reported PRN‐related symptoms, the patient was instructed to stop the PRN medication.

If the HF risk score remained in the high‐risk state for 14 days after the last day of the PRN intervention, the intervention pathway was repeated. No more than 3 PRN interventions were initiated over 90 days. If 3 PRN interventions occurred within 90 days, subsequent PRN interventions were suspended until the high‐risk status was resolved by alternate interventions by the investigator.

The investigator was notified by the MCMS nurse if the PRN intervention was initiated, if a high‐risk score met rule‐out criteria, or if a PRN intervention was stopped early due to safety. If 2 PRN interventions were implemented within 45 days or if 3 within 90 days, the investigator was required to take a clinical action (changes in baseline medication or PRN prescription or alternate therapeutic interventions) or provide a rationale for not taking a clinical action. Finally, if chronically low subcutaneous impedance was observed (ie, 60‐day mean of <600 Ω) indicating possible chronic fluid overload, the investigator was notified and requested to consider a change to baseline medications.

### Statistical Analysis

Data collected included baseline characteristics, medications, and AEs. Symptoms were also collected at the onset of high‐risk alert, initiation of PRN intervention, and the end of completed interventions. The reasons for rule‐out condition, if rule‐out criteria were satisfied, were collected. Weight and blood pressure measurements as well as a symptom questionnaire were collected during the PRN interventions. AEs were adjudicated by an independent clinical events committee. Data on the total number of high‐risk scores triggered during the intervention period, the proportion that were ruled out, the reason for the rule‐out, the total number of PRN interventions, and the occurrence of HF hospitalization and outpatient HF events were collected. The clinical events committee adjudicated inpatient or outpatient intervention for heart failure decompensation (HF event), defined as an event requiring invasive HF therapy (eg, intravenous or subcutaneous diuretics/vasodilators/inotropes) or ultrafiltration.

## Results

### Patient Characteristics

Phase 1 of the ALLEVIATE‐HF study enrolled a total of 63 patients from September 2020 to September 2021, of whom 59 were implanted with an ICM and were randomized (41 to the observation and 18 to the intervention arm). After 7 months of follow‐up in the observation arm, all 41 patients crossed over to the intervention arm per protocol. Interventions in all 59 patients (18 randomized to intervention and 41 randomized to observation who crossed over after 7 months to the intervention phase) are reported in this study. There were no SAEs related to the ICM or the implant procedure. There were 3 AEs related to the ICM system, of which 1 (device extrusion) was related to the implant. The baseline characteristics of the randomized patients are shown in Table 1. The mean left ventricular ejection fraction was 51%, 68% of patients had a left ventricular ejection fraction ≥50%, and 44% had New York Heart Association class II symptoms. Overall, 98% of participants were on diuretics and >80% had recent HF admissions or received outpatient intravenous diuretics. The most common comorbidities included hypertension (56 patients, 95%) and AF (33 patients, 56%). The mean follow‐up duration in the intervention period in both arms as of the data cutoff of March 17, 2023 was 11.8±8.1 months. Patients in the observation arm had a mean follow‐up of 6.3±1.9 months before exiting or crossover to the intervention period. There were 13 deaths during the study; none were adjudicated by the clinical events committee to be related to the procedure, device, or a PRN intervention. Seven patients received a device upgrade to a pacemaker or defibrillator device during the study and thus exited. There were 23 HF events (HFEs) during the study as adjudicated by the clinical events committee. During the intervention period, there were 0.023 HFEs per patient month for both arms (intervention arm for the entire follow‐up and observation arm for follow‐up after crossover) and 0.027 HFEs per patient month during the observation period before patients crossed over to the intervention period. HFE rates were likely biased due to the impact of COVID‐19, as the observation arm was predominantly followed during the pandemic, when HFE rates decreased, and the data accumulation for the intervention arm were mostly after COVID‐19.

**Table 1:** Baseline Characteristics of Patients Randomized in Phase 1 of ALLEVIATE‐HF Study

	All patients	Randomized to intervention	Randomized to observation
Number of patients	59	18	41
Age, y	68.2 ± 11.8	70.4 ± 11.9	67.2 ± 11.7
Male sex	35 (59.3%)	10 (55.6%)	25 (61.0%)
Symptomatic HF history for inclusion eligibility*			
Hospital admission for HF within the past 12 mo	39 (66.1%)	12 (66.7%)	27 (65.9%)
Intravenous HF therapy or ultrafiltration within the last 6 mo	9 (15.3%)	3 (16.7%)	6 (14.6%)
Elevated BNP/N‐terminal‐proBNP within the past 3 mo	11 (18.6%)	3 (16.7%)	8 (19.5%)
Clinical history			
NYHA classification class II	26 (44.1%)	7 (38.9%)	19 (46.3%)
NYHA classification class III	33 (55.9%)	11 (61.1%)	22 (53.7%)
LVEF	50.9 ± 12.6	51.8 ± 10.9	50.6 ± 13.3
Patients with LVEF ≥50%	40 (67.8%)	11 (61.1%)	29 (70.7%)
Hypertension	56 (94.9%)	17 (94.4%)	39 (95.1%)
Coronary artery disease	23 (38.9%)	4 (22.2%)	19 (46.3%)
Myocardial infarction	9 (15.3%)	2 (11.1%)	7 (17.1%)
Renal dysfunction	17 (28.8%)	6 (3.33%)	11 (28.2%)
Diabetes	34 (57.6%)	11 (61.1%)	23 (56.1%)
Sleep apnea	30 (50.8%)	9 (50.0%)	21 (51.2%)
Chronic obstructive pulmonary disease	20 (33.9%)	6 (33.3%)	14 (35.9%)
Secondary pulmonary hypertension	7 (11.9%)	4 (22.2%)	3 (7.3%)
Atrial fibrillation	33 (55.9%)	13 (72.2%)	20 (48.8%)
Heart failure cause: ischemic	11 (18.6%)	2 (11.1%)	9 (22.0%)
Medications			
Beta‐blocker	53 (89.8%)	17 (94.4%)	36 (87.8%)
Renin–angiotensin–aldosterone system inhibitor (angiotensin‐converting enzyme or angiotensin receptor blocker or ARNI)	40 (67.8%)	12 (66.7%)	28 (68.3%)
ARNI	8 (13.6%)	3 (16.7%)	5 (12.2%)
Mineralocorticoid receptor antagonist	18 (30.5%)	6 (33.3%)	12 (29.3%)
Sodium glucose transporter 2 inhibitor	4 (6.8%)	2 (11.1%)	2 (4.9%)
Diuretic	58 (98.3%)	17 (94.4%)	41 (100.0%)
Anticoagulant	34 (57.6%)	13 (72.2%)	21 (51.2%)
Antiarrhythmic	11 (18.6%)	6 (33.3%)	5 (12.2%)
Laboratory results			
Creatinine, mg/dL	1.2 ± 0.4	1.2 ± 0.4	1.2 ± 0.4
Serum urea nitrogen, mg/dL	23.0 ± 9.2	25.1 ± 10.9	22.3 ± 8.5
Estimated glomerular filtration rate, mL/min per 1.73 m^2^	54.2 ± 19.8	51.5 ± 13.5	55.6 ± 22.5
Sodium, mmol/L	138.8 ± 3.0	138.8 ± 2.7	138.8 ± 3.2
Potassium, mmol/L	4.1 ± 0.5	4.1 ± 0.5	4.1 ± 0.5

ALLEVIATE‐HF indicates Algorithm Using LINQ Sensors for Evaluation and Treatment of Heart Failure; ARNI, angiotensin receptor neprilysin inhibitor; BNP, B‐type natriuretic peptide; eGFR,; HF, heart failure; LVEF, left ventricular ejection fraction; and NYHA, New York Heart Association.

* Patient listed only in first category met.

### Rule‐Out Criteria

**Figure 2:**
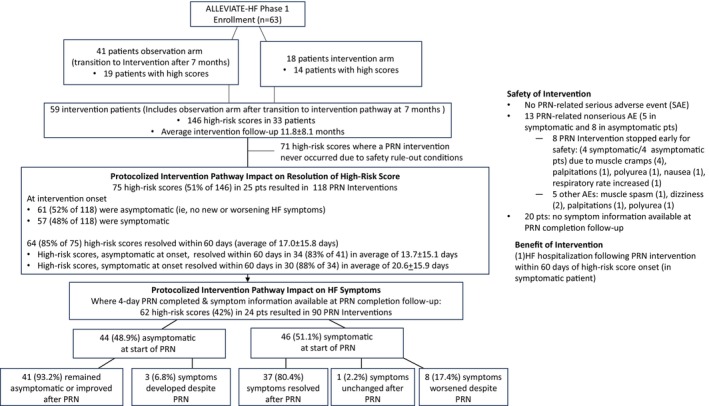
A diagram describing the breakdown of high‐risk score alerts, PRN interventions, and symptom resolution during the intervention period in Phase 1 of the ALLEVIATE‐HF study. AE indicates adverse event; ALLEVIATE‐HF, Algorithm Using LINQ Sensors for Evaluation and Treatment of Heart Failure; HF, heart failure; PRN, *pro re nata* or as needed; Pts, patients; and SAE, serious adverse event.

A total of 146 high‐risk score alerts occurred in 33 patients during the intervention period. Of these, a PRN intervention was implemented in 75 (51.4%). PRN interventions were withheld for 71 high‐risk score alerts due to rule‐out conditions (Figure 2). Of the 71 high‐score alerts where PRN interventions were withheld due to safety rule‐out conditions, 8 patients never received a PRN intervention.

### PRN Interventions and Other Medication and Therapeutic Changes

Medications prescribed by investigators at enrollment for PRN interventions mostly consisted of increasing the dosage of diuretics for 4 days (75% furosemide, 17% torsemide, 10% bumetanide, 6% metolazone). PRN prescriptions were changed 9 times in 8 patients during the study: 6 dose adjustments and 3 changes of diuretic type (1 from loop to thiazide diuretic and 2 changes to a different loop diuretic). The 75 high‐risk scores for which a PRN intervention was implemented were in 25 patients. The PRN was immediately initiated upon initial assessment in 48 high‐risk score alerts and initiated after a delay in 27 due to intervention pathway rule‐out conditions. Of the 75 high scores with PRN interventions, 43 received 1, 21 had 2, and 11 had 3 PRN interventions, resulting in a total of 118 PRN interventions (Table S2). The 4‐day course of PRN was completed in 103 interventions, with 8 stopped before completion due to PRN‐related AEs, and 7 where completion was unable to be confirmed. Of the 118 total PRN interventions that were initiated, 57 (48%) were symptomatic and 61 (52%) were asymptomatic (ie, had no new or worsening HF symptoms) at time of PRN initiation.

Following each PRN intervention, investigators were directed to assess and optimize the patient's baseline medication regimen based on current published guidelines, which were updated during the study.^25^, ^26^ After the 118 PRN interventions, a total of 76 medications were added or dosage modified within 30 days; 47 were targeted HF treatment (25 diuretics, 13 beta blockers, 4 renin–angiotensin–aldosterone system inhibitors, 1 sodium glucose transporter 2 inhibitors, 3 hydral‐nitrates, and 1 mineralocorticoid receptor antagonist) and 6 targeted cardiac arrhythmia management (3 anticoagulants and 3 antiarrhythmics) (Table S3). There were 4 patients in whom anticoagulation was initiated throughout the entire study duration and 5 patients in whom antiarrhythmics were initiated. Further, there were a total of 9 AF ablations and 5 cardioversions that were documented after subjects were enrolled in the study.

### Safety of PRN Interventions

There were no SAEs in Phase 1 that were related to PRN intervention and no Reveal LINQ system‐related or procedure related SAEs at 6 months. Given that there were 118 PRN interventions, the estimated CI for the rate of PRN related SAEs is 0 to 0.0254, with the upper confidence limit lower than the 5% specified cutoff defined for the Phase 2 of the study.

There were 13 non‐SAEs related to PRN, of which 8 resulted in the PRN interventions being stopped before the 4‐day completion as per protocol. Of these 8 interventions, there were muscle cramps in 4 cases, and 1 case each of palpitations, polyuria, nausea, and a high respiratory rate. Laboratory data were requested if PRN was discontinued early due to meeting safety thresholds; this was performed within 21 days of PRN intervention in 4 of the 8 cases where PRN intervention was stopped early. The laboratory results are summarized in Table 2. The other 5 AEs occurred after PRN intervention was completed and were due to dizziness (2 cases), muscle spasms, palpitations, and polyuria. During the safety monitoring of PRN interventions, the average weight loss from start to end of PRN intervention was 1.5 pounds.

**Table 2:** Laboratory Values at Baseline and After PRN Intervention in Cases Where PRN Intervention Was Stopped Early (N=4 Where Laboratory Test Was Completed Within 21 Days After Stopped PRN)

	At start of intervention period in patient with stopped PRN (N=4)		Within 21 d of stopped PRN intervention (N=4)	
	Mean±SD	[Min, Max]	Mean (std)	[Min, Max]
Serum urea nitrogen, mg/dL	24.0±16.7	[15–49]	25.3±13.6	[12–41]
Creatinine, mg/dL	1.3±0.4	[0.91–1.9]	1.5±0.7	[0.8–2.3]
Estimated glomerular filtration rate, mL/min per 1.73 m^2^	45.5±10.5	[36.0–59.9]	48.3±24.7	[20–74]
Sodium, mmol/L	137.5±3.0	[134–140]	138.0±3.7	[134–142]
Potassium, mmol/L	4.3±0.3	[3.8–4.5]	4.2±0.5	[3.6–4.7]

PRN, *pro re nata*.

### Symptoms and High‐Risk Resolution

Post PRN symptom information was available in 90 of 118 (76.3%) PRN interventions. In 8 cases, the PRN was stopped early and in another 20, follow‐up information was not available. In the 90 with available post‐day‐5 PRN intervention follow‐up, at the time of PRN initiation, 44 (49%) were asymptomatic compared with baseline and 46 (51%) were symptomatic. In patients with symptoms at PRN initiation, symptoms resolved in 37 (80.4%) after PRN and worsened or stayed the same despite PRN in 9 (19.6%). In asymptomatic patients, no symptoms developed after PRN in 41 (93.2%) of cases and worsening symptoms developed despite PRN in 3 cases (6.8%) (Figure 2). Two HF hospitalizations occurred within 60 days following a PRN intervention.

The high‐risk score status resolved (became low or medium risk) within 60 days from onset in 64 of the 75 (85%) with PRN interventions, with a mean resolution time of 17.0±15.8 days. The risk score was not expected to resolve for 14 to 21 days due to the latency or data aggregation time required to reflect the updated risk, even though the actual clinical event may have resolved in 3 to 4 days.^1^, ^15^ High‐risk alerts that were asymptomatic at onset resolved in 34 of 41 (83%) within 60 days, with a mean resolution time of 13.7±15.1 days, and high‐risk alerts with new or worsening HF symptoms at onset resolved within 60 days in 30 of 34 (88%) following PRN interventions, with a mean resolution time of 20.6±15.9 days.

### Characterization of HF Events During the Entire Study

There was a total of 23 HFEs in 15 patients, of which 6 HFEs in 6 patients were not preceded by any high risk in the 30 days before the HFE. There were 17 HFEs in 11 patients where the patient had days of high risk in the 30 days before HFE, with 14 being high risk on the day of the HFE. The high‐risk alert onset for these 17 were on average 166 days before the HFE. Of the 17 HFEs with high risk within 30 days before the HFE, 4 received PRN intervention within the past 60 days before the HFE, and 13 did not receive PRN within 60 days before HFE (7 received PRN more than 60 days before HFE and 6 did not receive any PRN due to rule‐out conditions for safety).

## Discussion

In Phase 1 of the ALLEVIATE‐HF trial, we demonstrated the safety of a personalized intervention pathway triggered by ICM high‐risk scores, irrespective of HF symptoms. There were no SAEs reported after implementing a total of 118 interventions triggered solely by alerts. Only 8 interventions did not complete the 4‐day diuretic intensification course due to safety concerns for symptoms commonly seen in the acute diuresis phase, such as polyuria, muscle cramps, and palpitations. Available assessment of serum electrolytes and renal function in patients whose interventions were stopped early did not show acute changes. Most notably, there were no safety concerns when interventions were applied to patients who were asymptomatic at intervention initiation. Interventions caused the resolution of worsening HF symptoms in most symptomatic patients (80%) and prevented HF symptoms in nearly all asymptomatic patients (93%).

These results address an important gap in remote monitoring management of HF by establishing a connection between alerts and action. They offer an early intervention strategy that can be safely implemented before symptoms occur. The excellent safety of the personalized intervention strategy described in this paper can be attributed to several reasons. First, the individualized nature of the intervention. Diuretic intensification prescription and safety rule‐out parameters such as blood pressure cutoffs, weight changes, and heart rate limits during AF, were determined by the site investigators who are most familiar with the patient history and prior responses and tolerance to diuretics. This personalized approach helped minimize adverse reactions and tailored intervention to each patient's needs. Second, the MCMS workflow ensured that interventions were applied in an actionable HF setting. This was achieved through a systematic rule‐out process of alternate clinical conditions that mimic HF (such as pneumonia, chronic obstructive pulmonary disease, etc.), trigger HF (AF), or make diuretics intensification ineffective or unsafe (end‐stage kidney disease, hypotension, inotrope dependency, use of selective pulmonary vasodilators, etc.). Conditions such as pneumonia or chronic obstructive pulmonary disease exacerbation alter components of the risk score through common physiological signals with HF (elevated respiratory rate, tachycardia, decreased physical activity) and can lead to elevation in risk scores. Treating these conditions with diuretic intensification delays appropriate management. This was overcome by the safety rule stepwise approach that enabled prompt detection of non‐HF conditions, alerting investigators, and facilitating suitable treatments. Third, ensuring adherence to medical therapy before intervention was applied. Medication nonadherence is a common cause of HF exacerbations and readmissions. The workflow included a comprehensive process that assesses recent medication changes made at outpatient visits or posthospital discharges. Nearly half of the PRN intervention suspension was due to recent medication changes, underscoring the importance of proper reconciliation of background medical therapy before safely implementing a diuretic intervention. Fourth, executing remote safety threshold monitoring during and following PRN intervention by certified HF nurses. Half of all high‐risk scores did not receive interventions due to meeting rule‐out conditions, which is high but not surprising given rule‐out conditions are common among patients with HF and in this study cohort, where 56% had a history of AF and 34% chronic obstructive pulmonary disease. The risk score is a dynamic variable and many patients became intervention eligible once the rule‐out condition resolved. In fact, 36% of all interventions were delayed after initial temporary suspensions.

As a result, our strategy identified high‐risk patients and improved overall HF management. Notifications sent to investigators resulted in 76 medication changes, including 47 medications specifically targeted for HF management (Table S2). The medication classes used can be explained by the dominant preserved ejection fraction HF type among the study participants (68% with an ejection fraction >50%), who were mostly enrolled before sodium glucose transporter 2 inhibitors were included in current guidelines.^26^ The study interventions were not limited to diuretic intensifications but included further steps toward the optimization of HF management. Patients who require >3 PRN interventions within 90 days and those with persistently low tissue impedance represent a high‐risk cohort. Previous data on transthoracic impedance have shown that chronic low values may suggest resistance to medication interventions.^27^ Faced with these circumstances, the study protocol required investigators' notifications to determine further steps.

Our data showed that the safety of the intervention was seen in symptomatic and asymptomatic patients alike. Data collected regarding symptoms at the initiation of PRN and the follow‐up after PRN completion showed that 51% of patients experienced worsening symptoms at PRN initiation. Among the symptomatic patients, >80% experienced resolution of their worsening symptoms, whereas 17% experienced further worsening despite the PRN intervention. In contrast, among the 44 patients without worsening symptoms at PRN initiation compared with baseline, only 7% developed worsening symptoms, whereas 93% either remained without worsening symptoms or reported improved status compared with baseline. Although not powered for comparative analysis, these findings reemphasize that PRN interventions solely based on high‐risk scores were successful in preventing worsening HF. It also highlights the significance of early intervention once a high‐risk score is detected, aligning with previously published data.^16^, ^18^

In most patients, the implementation of medication intervention resulted in the resolution of high‐risk scores on an average of 17 days. This adds further validation and establishes a cause‐and‐effect relationship between actionable HF management and recovery of high HF risk score.^15^ Notably, the resolution of high‐risk scores was observed in both symptomatic and asymptomatic patients to nearly similar extent. The time lag between the improvement of HF symptoms and the recovery of the high‐risk score is due to variations in the rate at which each risk score individual parameter recovers and the 7‐day and 30‐day aggregation methods used for the generation of the risk score.^1^

In previous trials, enhanced decongestion strategies did not lead to improved long‐term HF outcomes and had minimal effects on HF symptoms.^28^ However, most of these trials were carried out in inpatient settings during periods of heightened neurohormonal activations. In contrast, our study focused on patients at risk of worsening HF in an outpatient setting, who were identified early based on the detection of HF triggers like AF burden, compensatory physiological mechanisms such as heart rate and heart rate variability, and early signs of volume retention through changes in bioimpedance. Early intervention in this context has been shown to improve HF outcomes. A recent meta‐analysis involving 1350 patients with HF revealed that remote hemodynamic monitoring‐based management led to a significant decrease in HF hospitalizations with a hazard ratio of 0.64 (95% CI, 0.55–0.76); *P*<0.0001 and a significant reduction in overall mortality with a hazard ratio of 0.75 (95% CI, 0.57–0.99); *P*=0.043.^29^ Our study protocol involves measures aimed at adjusting guideline‐directed medical therapies, particularly in patients with consistently high‐risk scores or chronic low impedance.

The ability of ICMs to detect arrhythmias accurately offers several additional advantages in HF management. AF is a significant factor that can lead to the acute decompensation of HF, and it is also a common complication of worsening HF. When AF was identified as a contributor to the high‐risk score, the site investigators were alerted if new‐onset or ventricular rate exceeded a safety threshold. The safety protocol allows the primary investigator to either withhold PRN intervention if AF is believed to be the primary trigger for worsening HF or to allow PRN intervention if worsening HF is considered the trigger for AF. Uncovering subclinical AF, determining the need for systemic anticoagulation, initiating antiarrhythmic drugs, or referring the patient for rhythm control interventions are added advantages to the ICM HF management strategy (see Table S2 with detailed medication changes). ICMs also allow device‐based therapy optimization through the detection of ventricular arrhythmias or conduction system abnormalities. Our data showed that within an average of 11 months of follow‐up, 12% of study patients underwent appropriate upgrades to either pacemakers or implantable cardioverter‐defibrillators based on data received through ICM transmissions, similar to previous reports.^8^

The primary purpose of ALLEVIATE‐HF Phase 1 was to investigate safety and not to determine the effectiveness of personalized intervention pathways in improving HF outcomes. The efficacy of this approach will be thoroughly assessed in an ongoing larger, blinded, randomized, and controlled Phase 2 of the ALLEVIATE‐HF study (NCT04452149).

To summarize, an effective strategy for managing remote HF should consider several key interrelated actions. These actions include (1) validating the technology used for HF sensors, ensuring their accuracy and reliability; (2) promptly recognizing and responding to high‐risk alerts regardless of symptom status; (3) tailoring therapeutic interventions to each patient based on their response to treatment; (4) conducting thorough patient screening to rule out conditions that mimic HF; and (5) regularly assessing daily biometrics, weight, and symptoms before and after medication interventions to ensure safety. In the initial phase of the ALLEVIATE‐HF trial, we have demonstrated the feasibility and safety of implementing such an integrated approach.

### Limitations

The current study had limitations in terms of sample size, as it enrolled only 63 patients. However, the ongoing Phase 2 of the ALLEVIATE‐HF study plans to enroll a larger number of patients, exceeding 650. Another limitation was the absence of an appropriately powered control arm comparison, but this issue will be addressed in Phase 2. The first part of the study took place during the COVID‐19 pandemic which may have affected event rates and the collection of certain data.

The ALLEVIATE‐HF program used centralized HF services with nursing staff experienced in remote HF management and ICM device data. The study workflow can be easily implemented by any HF center, provided that the HF nursing staff receives proper training.

### Conclusions

The findings from the ALLEVIATE‐HF Phase 1 trial support the safety of a personalized medication intervention strategy, based on a HF risk score generated from sensors in an ICM, implemented by a centralized nurse service according to a protocolized workflow that integrates predetermined investigator‐directed dosing and safety parameters. These data offer a highly anticipated framework that enhances the field of remote monitoring for HF by connecting risks to actionable HF interventions, without depending on symptoms, while guaranteeing safety through the appropriate selection of intervention eligibility, ongoing evaluation of risk status, and determining the necessity for repetitive interventions based on predefined and personalized safety parameters.

## Sources of Funding

This study was funded by Medtronic.

## Disclosures

Rami Kahwash has served as consultant to Medtronic, Cardionomic, Edwards Lifesciences, Impulse Dynamics, and scPharmaceuticals. Michael R. Zile has served as a consultant to Medtronic. Barry Bertolet has served as a consultant to Medtronic. Laura Gravelin has served on advisory boards for Medtronic Muhammad Shahzeb Khan serves on an advisory board for Bayer. Jennifer Wehking, Brian Van Dorn, Shantanu Sarkar, Verla Laager, Bart Gerritse, and Aimee Laechelt are Medtronic employees and shareholders. Javed Butler has served as a consultant for Medtronic, Abbott, American Regent, Amgen, Applied Therapeutic, AskBio, Astellas, AstraZeneca, Bayer, Boehringer Ingelheim, Boston Scientific, Bristol Myers Squibb, Cardiac Dimension, Cardiocell, Cardior, CSL Bearing, CVRx, Cytokinetics, Daxor, Edwards, Element Science, Faraday, Foundry, G3P, Innolife, Impulse Dynamics, Imbria, Inventiva, Ionis, Lexicon, Lilly, LivaNova, Janssen, Merck, Occlutech, Owkin, Novartis, Novo Nordisk, Pfizer, Pharmacosmos, Pharmain, Prolaio, Regeneron, Renibus, Roche, Salamandra, Sanofi, SC Pharma, Secretome, Sequana, SQ Innovation, Tenex, Tricog, Ultromics, Vifor, and Zoll.Prasad Chalasani has no disclosures to report.

## Supporting information

Data S1
Tables S1–S3
