# Supplementary material for: Personalized Intervention Strategy Based on a Risk Score Generated From Subcutaneous Insertable Cardiac Monitor: Results From Phase 1 of ALLEVIATE‐HF
Source: J Am Heart Assoc. 2024 Oct 11;13(20):e035501. doi: 10.1161/JAHA.124.035501 (PMC11935577; doi:10.1161/JAHA.124.035501)
Supplement: Supplementary file 1 — Data S1 Tables S1–S3 [file JAH3-13-e035501-s001.pdf]

SUPPLEMENTAL MATERIAL

ALLEVIATE-HF Phase I Investigators:

From Baptist Health, Jacksonville Beach, FL, USA: Dr. Ruple Galani; Bay Area Cardiology Associates PA, Brandon, FL, USA: Dr. Stephen Mester; Cardiology Consultants of Philadelphia, Philadelphia, PA, USA: Dr. Matthew Goldstein; Cone Health, Greensboro, NC, USA: Dr. James Allred; Doylestown Health Cardiology, Doylestown, PA, USA: Dr. Renee Sangrigoli; Iowa Heart Center. Des Moines, IA, USA: Dr. Jennifer Goerbig-Campbell; Medical University of South Carolina, Charleston, SC, USA: Dr. Gregory Jackson; North Memorial Health Heart & Vascular Center, Robbinsdale, MN, USA: Dr. Chike Obi; Saint Joseph's Medical Center, St Paul, MN, USA: Dr. Cyrus Buhari; The Stern Cardiovascular Foundation, Memphis, TN, USA: Dr. Frank McGrew; Tyler Cardiovascular Consultants, Tyler, TX, USA: Dr. Raul Torres; Virtua Lourdes Cardiology Services, Cherry Hill, NJ, USA: Darius Sholevar.

Data S1.

Supplemental Methods

Medtronic Care Management Services (MCMS) Role in Study Framework

Medtronic Care Management Services (MCMS) employed licensed registered nurses (RNs) for the ALLEVIATE-HF study. After enrollment, the investigator provided the following personalized intervention plan for each individual patient:

- PRN prescription to be implemented by MCMS nurse on high-risk alert after rule-out condition evaluation.
- Safety thresholds for blood pressure changes and weight loss during PRN for discontinuation of PRN medication during 4-day PRN implementation.
- If patient has known history of AF, the threshold for ventricular rate during atrial fibrillation (VRAF) at which the MCMS nurse should consult the investigator prior to initiating a PRN.

MCMS provided patients with remote monitoring telehealth equipment to collect and transmit data (e.g. blood pressure and weight) and symptoms. Patients in both arms were encouraged to transmit weight and blood pressure daily and respond to "YES/NO" health or symptom questions on the interview tablet, although this was only required during PRN.

Patients in both arms were requested to sleep near their MyCareLink Monitor to enable automatic nightly transmissions. MCMS nurses monitored and mitigated possible reasons for missed transmissions from patients along with help from the technical team.

<u>Intervention Pathway</u>

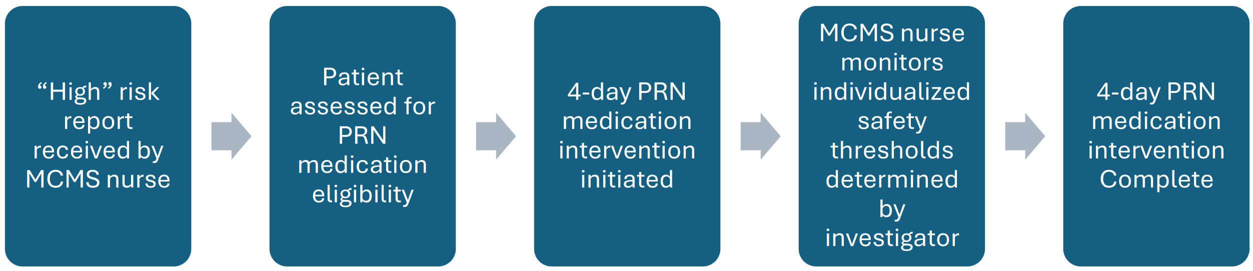

A. High Risk report received by MCMS Nurse

Patients in the intervention arm with a "high" risk status who had completed the 6-week device maturation period and had not undergone device repositioning in the past 6 weeks were assessed

by the MCMS nurse to confirm PRN eligibility. A minimum of 14 days following the last day of the patient's most recent PRN medication intervention (as applicable) must have passed prior to the MCMS nurse initiating the eligibility assessment process in relation to a current "high" risk status. The MCMS nurse reviewed the heart failure report including the patient's current overall risk status, the risk status for each of the individual diagnostic parameters, and trends of diagnostic and risk status data collected via CareLink.

B. MCMS nurse assessed PRN eligibility prior to assessing patient:

- Assess if AF is contributing to the high-risk report. If AF is contributing and the patient has no known history of AF or if known AF history and VRAF thresholds are met, the Investigator is notified to make a decision on how to proceed with patient by either proceeding with PRN assessment or delaying for the Investigators to take action to address the AF (i.e. medication change, cardioversion, and/or ablation).
- Confirm patient has not undergone 3 rounds of PRN within the past 90 days.

If any of the above conditions were met, the PRN eligibility assessment did not continue and the Investigator was provided a copy of the heart failure risk report and was informed of the reason patient was not eligible for PRN. If 3 PRNs were given in a 90-day period, Investigators were requested to evaluate for guideline directed medical therapy (GDMT) and no new PRN was initiated until the current high-risk status was resolved.

C. MCMS nurse contacted patient to assess for PRN eligibility (Rule Out Criteria):

- Pulmonary hypertension, as evidenced by use of/new prescription for pulmonary hypertension agents
- HF Class medication change within the past 14 days
- Baseline diuretic change within the past 14 days
- Non-adherence to baseline diuretic (e.g. inability to fill prescription, etc.)
- COPD care plan change in the past 7 days
- Hospital discharge in past 7 days
- Evidence of febrile illness in past 48 hours
- Systolic blood pressure < 85 mmHg on day of contact with new hypotensive symptoms (If blood pressure not available, verbally assessed for new hypotensive symptoms)

If any of the conditions were met, the PRN was not initiated and the MCMS nurse suspended the eligibility assessment process for 7-14 days and the Investigator was provided a copy of the heart failure risk report and the reason for rule-out. MCMS provided additional notification in cases where 2 PRN interventions were initiated in 45 days or the 60-day mean was < 600 ohms. If the

risk status was “high” following the suspension period, the evaluation process was restarted from the beginning.

D. The 4-day PRN medication intervention

The MCMS nurse requested the patient to initiate the investigator-prescribed PRN medication if none of the rule-out criteria were met. The MCMS nurse notified the investigator that the PRN had been initiated and provided the Investigator a copy of the heart failure risk report. Investigators were requested to review the patient’s baseline medications and optimize medications according to guideline directed medical therapy (GDMT) when appropriate.

E. MCMS nurse monitored individualized safety thresholds determined by the investigator.

Patients were asked to provide daily blood pressure and weight measurements throughout the 4-day PRN. If blood pressure and weight measurements were not provided, the MCMS nurse contacted the patient via phone to verbally collect measurements or perform an adverse symptom questionnaire. If the safety thresholds were met, the MCMS nurse contacted the patient to evaluate for safety symptoms (e.g. new lightheadedness when moving from sitting to standing and/or muscle cramps or charley horses). If these symptoms were present, the MCMS nurse instructed the patient to discontinue the PRN medication and the investigator was notified. Labs were required to be drawn within 7 days of the PRN medication being stopped due to safety.

F. 4-Day PRN Medication Intervention complete.

Following the PRN intervention, the MCMS nurse would wait for a minimum of 14 days prior to repeat evaluation of the patient eligibility assessment for any ongoing or new “high” risk status.

Table S1. Inclusion and exclusion criteria for Phase 1 of ALLEVIATE-HF study.

INCLUSION CRITERIA	18 years of age or older
	Life expectancy of 12 months or more
	Willing and able to provide written informed consent
	Willing and able to comply with the protocol, including follow-up visits and Carelink transmissions.
	NYHA Class II/III
	HF event (HF event defined as meeting any one of the following three criteria):<br>1. Admission with primary diagnosis of HF within the last 12 months, OR<br>2. Intravenous HF therapy (e.g., IV diuretics/vasodilators) or ultrafiltration within the last 6 months, OR<br>3. Within the last 3 months: EF > 50%, then BNP> 150 or NTpro-BNP > 450 OR If EF is <50%, then BNP>300 or NTpro-BNP > 900
EXCLUSION CRITERIA	Pregnant (all females of child-bearing potential must have a negative pregnancy test within 1 week of enrollment)
	Severe valve stenosis on echocardiogram
	Enrolled in another interventional study
	Existing IPG, ICD, CRT-D or CRT-P device or hemodynamic monitor
	Severe renal impairment (eGFR < 30 mL/min)
	Patient is on chronic renal dialysis
	Systolic blood pressure of < 90 mmHg at enrollment
	Chronic intravenous inotropic drug therapy
	MI or PCI/CABG within past 90 days
	Temporary or permanent mechanical circulatory support
	Heart transplant or is currently on heart transplant list
	Primary pulmonary hypertension (pre-capillary, WHO group 1,3,4,5)
	Unable to undergo one round of PRN medication intervention (i.e. 4 days of increased diuretics dose), based on the judgement of the investigator (e.g. known history of side effects or intolerance to PRN dosing of diuretics)
	Liver disease, defined as AST/ALT > 5x normal, or bilirubin >2x normal
	Serum albumin < 3 g/dL
	Hypertrophic obstructive cardiomyopathy, constrictive pericarditis, or amyloidosis
	Complex adult congenital heart disease
	Active cancer involving chemotherapy and/or radiation therapy
	Weighs > 500 pounds

Table S2. **PRN Intervention Dosages.**

Patient	Baseline Diuretic*	PRN Intervention at time of High Risk**
1	FUROSEMIDE (80 mg QD AM)	FUROSEMIDE (20 mg QD)
1	FUROSEMIDE (80 mg QD AM)	FUROSEMIDE (20 mg QD)
1	FUROSEMIDE (80 mg QD AM)	FUROSEMIDE (20 mg QD)
2	FUROSEMIDE (80 mg QD)	FUROSEMIDE (80 mg QD)
2	FUROSEMIDE (80 mg QD)	FUROSEMIDE (40 mg QD)
3	FUROSEMIDE (40 mg QD)	FUROSEMIDE (40 mg QD)
3	FUROSEMIDE (40 mg QD)	FUROSEMIDE (40 mg QD)
3	FUROSEMIDE (40 mg QD)	FUROSEMIDE (40 mg QD)
4	TORSEMIDE (20 mg BID)	TORSEMIDE (20 mg QD)
4	TORSEMIDE (20 mg BID)	TORSEMIDE (20 mg QD)
4	TORSEMIDE (20 mg BID)	TORSEMIDE (20 mg QD)
4	TORSEMIDE (20 mg BID)	TORSEMIDE (20 mg QD)
4	TORSEMIDE (20 mg BID)	TORSEMIDE (20 mg QD)
4	TORSEMIDE (20 mg BID)	TORSEMIDE (20 mg QD)
4	TORSEMIDE (20 mg BID)	TORSEMIDE (20 mg QD)
5	TORSEMIDE (10 mg QD)	TORSEMIDE (20 mg QD)
5	TORSEMIDE (10 mg QD)	TORSEMIDE (20 mg QD)
5	TORSEMIDE (10 mg QD)	TORSEMIDE (20 mg QD)
5	TORSEMIDE (10 mg QD)	TORSEMIDE (10 mg QD)
5	TORSEMIDE (10 mg QD)	TORSEMIDE (10 mg QD)
6	TORSEMIDE (40 mg QD PM) & METOLAZONE (2.5 mg QD)	METOLAZONE (5 mg QD)
6	TORSEMIDE (40 mg QD PM) & METOLAZONE (2.5 mg QD)	METOLAZONE (5 mg QD)
6	TORSEMIDE (40 mg QD PM) & METOLAZONE (2.5 mg QD)	METOLAZONE (5 mg QD)
6	TORSEMIDE (40 mg QD PM) & METOLAZONE (2.5 mg QD)	METOLAZONE (5 mg QD)
6	TORSEMIDE (40 mg QD PM) & METOLAZONE (2.5 mg QD)	METOLAZONE (5 mg QD)
7	FUROSEMIDE (40 mg PRN)	LASIX (FUROSEMIDE) (40 mg QD)
7	FUROSEMIDE (40 mg PRN)	LASIX (FUROSEMIDE) (40 mg QD)
7	FUROSEMIDE (40 mg PRN)	LASIX (FUROSEMIDE) (40 mg QD)
7	FUROSEMIDE (40 mg PRN)	LASIX (FUROSEMIDE) (20 mg QD)
7	FUROSEMIDE (40 mg PRN)	LASIX (FUROSEMIDE) (20 mg QD)
7	FUROSEMIDE (40 mg PRN)	LASIX (FUROSEMIDE) (20 mg QD)
8	FUROSEMIDE (20 mg QD)	LASIX (20 mg QD)
8	FUROSEMIDE (20 mg QD)	LASIX (20 mg QD)
9	TORSEMIDE (40 mg BID)	TORSEMIDE (20 mg QD)
9	TORSEMIDE (40 mg BID)	TORSEMIDE (20 mg QD)
9	TORSEMIDE (40 mg BID)	TORSEMIDE (20 mg QD)

10	FUROSEMIDE (20 mg QD)	FOUROSEMIDE (20 mg QD)
10	FUROSEMIDE (20 mg QD)	FOUROSEMIDE (20 mg QD)
11	TORSEMIDE (40 mg BID) & METOLAZONE (2.5 mg 1 TIME PER WEEK)	METOLAZONE (2.5 mg QD)
11	TORSEMIDE (40 mg BID) & METOLAZONE (2.5 mg 1 TIME PER WEEK)	METOLAZONE (2.5 mg QD)
11	TORSEMIDE (40 mg BID) & METOLAZONE (2.5 mg 1 TIME PER WEEK)	METOLAZONE (2.5 mg QD)
11	TORSEMIDE (40 mg BID) & METOLAZONE (2.5 mg 1 TIME PER WEEK)	METOLAZONE (2.5 mg QD)
11	TORSEMIDE (40 mg BID) & METOLAZONE (2.5 mg 1 TIME PER WEEK)	METOLAZONE (2.5 mg QD)
12	FUROSEMIDE (40 mg QD)	LASIX (40 mg QD)
12	FUROSEMIDE (40 mg QD)	LASIX (40 mg QD)
12	FUROSEMIDE (40 mg QD)	LASIX (40 mg QD)
12	FUROSEMIDE (40 mg QD)	LASIX (40 mg QD)
12	FUROSEMIDE (40 mg QD)	LASIX (40 mg QD)
12	FUROSEMIDE (40 mg QD)	LASIX (40 mg QD)
12	FUROSEMIDE (40 mg QD)	LASIX (40 mg QD)
12	FUROSEMIDE (40 mg QD)	LASIX (40 mg QD)
12	FUROSEMIDE (40 mg QD)	LASIX (40 mg QD)
12	FUROSEMIDE (40 mg QD)	LASIX (40 mg QD)
12	FUROSEMIDE (40 mg QD)	LASIX (40 mg QD)
12	FUROSEMIDE (40 mg QD)	BUMEX (1 mg QD)
12	FUROSEMIDE (40 mg QD)	BUMEX (1 mg QD)
12	FUROSEMIDE (40 mg QD)	BUMEX (1 mg QD)
13	BUMETANIDE (1 mg QD)	BUMETANIDE (0.5 mg QD)
14	LASIX (40 mg BID) & HCTZ (25 mg QD)	LASIX (40 mg QD)
14	LASIX (40 mg BID) & HCTZ (25 mg QD)	LASIX (40 mg QD)
14	LASIX (40 mg BID) & HCTZ (25 mg QD)	LASIX (40 mg QD)
14	LASIX (40 mg BID) & HCTZ (25 mg QD)	LASIX (40 mg QD)
14	LASIX (40 mg BID) & HCTZ (25 mg QD)	LASIX (40 mg QD)
14	LASIX (40 mg BID) & HCTZ (25 mg QD)	LASIX (40 mg QD)
14	LASIX (40 mg BID) & HCTZ (25 mg QD)	FUROSEMIDE (40 mg QD)
15	LASIX (40 mg QD) & LOSARTAN/HCTZ (100/12.5 mg QD)	LASIX (40 mg QD)
15	LASIX (40 mg QD) & LOSARTAN/HCTZ (100/12.5 mg QD)	LASIX (40 mg QD)
15	LASIX (40 mg QD) & LOSARTAN/HCTZ (100/12.5 mg QD)	LASIX (40 mg QD)
16	FUROSEMIDE (40 mg QD)	FUROSEMIDE (40 mg QD)

16	FUROSEMIDE (40 mg QD)	FUROSEMIDE (40 mg QD)
17	LASIX (40 mg PRN)	FUROSEMIDE (40 mg QD)
17	LASIX (40 mg PRN)	FUROSEMIDE (40 mg QD)
17	LASIX (40 mg PRN)	FUROSEMIDE (40 mg QD)
17	LASIX (40 mg PRN)	FUROSEMIDE (40 mg QD)
17	LASIX (40 mg PRN)	FUROSEMIDE (40 mg QD)
17	LASIX (40 mg PRN)	FUROSEMIDE (40 mg QD)
18	BUMEX (2 mg BID)	BUMEX (2 mg QD)
18	BUMEX (2 mg BID)	BUMEX (2 mg QD)
18	BUMEX (2 mg BID)	BUMEX (2 mg QD)
18	BUMEX (2 mg BID)	BUMEX (2 mg QD)
18	BUMEX (2 mg BID)	BUMEX (2 mg QD)
18	BUMEX (2 mg BID)	BUMEX (2 mg QD)
18	BUMEX (2 mg BID)	BUMEX (2 mg QD)
18	BUMEX (2 mg BID)	BUMEX (2 mg QD)
18	BUMEX (2 mg BID)	BUMEX (2 mg QD)
18	BUMEX (2 mg BID)	BUMEX (2 mg QD)
18	BUMEX (2 mg BID)	BUMEX (2 mg QD)
19	FUROSEMIDE (20 mg QD)	LASIX (40 mg QD)
19	FUROSEMIDE (20 mg QD)	LASIX (40 mg QD)
19	FUROSEMIDE (20 mg QD)	LASIX (40 mg QD)
19	FUROSEMIDE (20 mg QD)	LASIX (40 mg QD)
20	FUROSEMIDE (40 mg BID)	LASIX (40 mg QD)
20	FUROSEMIDE (40 mg BID)	LASIX (40 mg QD)
21	FUROSEMIDE (40 mg QD)	FUROSEMIDE (40 mg QD)
21	FUROSEMIDE (40 mg QD)	FUROSEMIDE (40 mg QD)
22	FUROSEMIDE (40 mg 3 TIMES PER WEEK)	FUROSEMIDE (40 mg QD)
22	FUROSEMIDE (40 mg 3 TIMES PER WEEK)	FUROSEMIDE (20 mg QD)
23	TORSEMIDE (20 mg QOD)	TORSEMIDE (20 mg OTHER)
23	TORSEMIDE (20 mg QOD)	TORSEMIDE (20 mg QOD)
23	TORSEMIDE (20 mg QOD)	TORSEMIDE (20 mg QOD)
23	TORSEMIDE (20 mg QOD)	TORSEMIDE (20 mg QOD)
23	TORSEMIDE (20 mg QOD)	TORSEMIDE (10 mg QOD)
23	TORSEMIDE (20 mg QOD)	TORSEMIDE (10 mg QOD)
23	TORSEMIDE (20 mg QOD)	TORSEMIDE (10 mg QOD)
23	TORSEMIDE (20 mg QOD)	TORSEMIDE (10 mg QOD)
23	TORSEMIDE (20 mg QOD)	TORSEMIDE (10 mg QOD)
23	TORSEMIDE (20 mg QOD)	TORSEMIDE (10 mg QOD)
23	TORSEMIDE (20 mg QOD)	TORSEMIDE (10 mg QOD)
24	FUROSEMIDE (20 mg QD)	FUROSEMIDE (20 mg QD)
24	FUROSEMIDE (20 mg QD)	FUROSEMIDE (20 mg QD)

24	FUROSEMIDE (20 mg QD)	FUROSEMIDE (20 mg QD)
24	FUROSEMIDE (20 mg QD)	FUROSEMIDE (20 mg QD)
24	FUROSEMIDE (20 mg QD)	FUROSEMIDE (20 mg QD)
24	FUROSEMIDE (20 mg QD)	FUROSEMIDE (20 mg QD)
24	FUROSEMIDE (20 mg QD)	FUROSEMIDE (20 mg QD)
24	FUROSEMIDE (20 mg QD)	FUROSEMIDE (20 mg QD)
24	FUROSEMIDE (20 mg QD)	FUROSEMIDE (20 mg QD)
25	FUROSEMIDE (20 mg QD)	TORSEMIDE (20 mg QD)

*Baseline diuretic at time of enrollment (does not reflect any modifications made throughout trial duration)

**4-day PRN medication intervention is incremental to baseline diuretic

Table S3. Medications Added or Dosage Modified within 30 days following PRN.

Antiarrhythmic	3	
Anticoagulant	3	
Anti-Diabetic Agent-GLP-1 Agonist	2	
Anti-Diabetic Agent-Insulin	4	
Anti-Diabetic Agent-SGLT2 Inhibitor	1	1
Anti-Hypertensive-Alpha2 Antagonist	1	
Anti-Hypertensive-Nitrate	2	2
Anti-Hypertensive-Vasodilator	1	1
Beta-Blocker	13	13
Calcium Channel Blocker	7	
Diuretic-Loop	23	23
Diuretic-Thiazide	1	1
Diuretic-Thiazide-Like	1	1
Lipid Lowering Agent-Non-Statin	2	
MRA-Nonsteroidal	1	1
MRA-Steroidal	2	
RAAS Inhibitor-ARB	3	3
RAAS Inhibitor-ARNI	1	1
Others / non-cardiac	5	
	71 Total Medication Changes	47 Total HF Medication Changes
